# Automated detection of discourse segment and experimental types from the text of cancer pathway results sections

**DOI:** 10.1093/database/baw122

**Published:** 2016-08-31

**Authors:** Gully A.P.C. Burns, Pradeep Dasigi, Anita de Waard, Eduard H. Hovy

**Affiliations:** 1 Information Sciences Institute, Viterbi School of Engineering, University of Southern California, Marina del Rey, CA 90292, USA; 2 Carnegie Mellon University, Language Technologies Institute, 5000 Forbes Avenue, Pittsburgh, PA 15213, USA; 3 Elsevier Research Data Services, Jericho, VT 05465, USA

## Abstract

Automated machine-reading biocuration systems typically use sentence-by-sentence information extraction to construct meaning representations for use by curators. This does not directly reflect the typical discourse structure used by scientists to construct an argument from the experimental data available within a article, and is therefore less likely to correspond to representations typically used in biomedical informatics systems (let alone to the mental models that scientists have). In this study, we develop Natural Language Processing methods to locate, extract, and classify the individual passages of text from articles’ Results sections that refer to experimental data. In our domain of interest (molecular biology studies of cancer signal transduction pathways), individual articles may contain as many as 30 small-scale individual experiments describing a variety of findings, upon which authors base their overall research conclusions. Our system automatically classifies discourse segments in these texts into seven categories (fact, hypothesis, problem, goal, method, result, implication) with an *F*-score of 0.68. These segments describe the essential building blocks of scientific discourse to (i) provide context for each experiment, (ii) report experimental details and (iii) explain the data’s meaning in context. We evaluate our system on text passages from articles that were curated in molecular biology databases (the Pathway Logic Datum repository, the Molecular Interaction MINT and INTACT databases) linking individual experiments in articles to the type of assay used (coprecipitation, phosphorylation, translocation etc.). We use supervised machine learning techniques on text passages containing unambiguous references to experiments to obtain baseline F1 scores of 0.59 for MINT, 0.71 for INTACT and 0.63 for Pathway Logic. Although preliminary, these results support the notion that targeting information extraction methods to experimental results could provide accurate, automated methods for biocuration. We also suggest the need for finer-grained curation of experimental methods used when constructing molecular biology databases

## Introduction/related work

The stated goal of the ‘Big Mechanism’ program of the US Defense Advanced Research Projects Agency (DARPA) is ‘to develop technology to help humanity assemble its knowledge into causal, explanatory models of complicated systems’ (http://www.darpa.mil/program/big-mechanism). The program is split into three technical areas: (i) ‘Reading’ (where information is automatically extracted from natural language text and tables used in published articles into a structured data format), (ii) ‘Assembly’ (where the structured information provided by the reading is organized and prioritized) and (iii) ‘Reasoning’ (where the assembled information is synthesized into functional knowledge about the domain of interest, using human-curated models and/or simulations). Since the program’s specified domain of investigation is molecular signaling pathways involved in cancer, this work has clear relevance to next-generation bioinformatics research. In particular, ‘Reading’ work performed within this program could be of direct significance to the field of biocuration.

Our work focuses on addressing this grand challenge using the following strategy: first, we develop models of the experimental methods and data used to justify interpretations instead of focusing on just the interpretations themselves (1), and second, we use the typical structures of biological discourse in articles to identify the textual elements concerned with observational and interpretive information. Our goal is to automate the construction of a database of experimental findings extracted from experimental research articles concerned with cancer pathways, in which the experimental status of the contents is explicitly recorded.

We next discuss the two principal descriptive dimensions of the work: formal representations of signaling pathways and discourse analysis.

### Knowledge representation of cellular signaling pathways

Computational models of biomedical mechanisms are generally formulated to represent interactions between species of molecules under a range of different methods, depending on the type of computation used to describe the underlying mechanisms. High-level descriptive languages such as the ‘Systems-Biology Markup Language’ (2), BioPax (3) or the ‘Biological Expression Language’ (4) provide a semantic representation of pathways, reactions and reactants (with encodings for additional information such as genetic details, post-translational modifications, reaction kinetics etc.). These languages provide interpretable summaries of pathway mechanisms that can be read by humans and/or reasoned about by computational knowledge representation and reasoning methods. For example, (5) use ‘perturbation biology’ experiments in cancer cells to systematically construct cell-type-specific signaling pathway models in conjunction with centralized pathway resources to nominate upstream–downstream drug combinations to combat drug resistance in melanoma. At a deeper level of description, executable languages such as Pathway Logic (PL) (6), Kappa (7), and PySB (8) provide simulation/reasoning frameworks that can make theoretical predictions about aspects of the state of the system under different hypothesized conditions (9). These types of formulations act as the target for reading systems and for the practice of biocuration generally.

However, automated curation methods that directly populate these types of representations focus primarily on the articles’ explicit findings or conclusions and largely overlook the underlying experimental evidence used to construct pathway representations (1). Often, these propositions are derived with little or no information about how they were ascertained experimentally. In practice, scientists pay a lot of attention to the derivation of a proposition before accepting it as fact. We therefore attempt to construct a well-founded reading methodology for molecular biology articles that automatically extracts not only such conceptual propositions that are based on experiments, but widens the focus of the extraction process to identify relevant experimental text, classify the type of experiment being performed and extract information relevant to both the experimental procedures/observations and their interpretations. Our view is motivated by previous work in building knowledge representations of data derived from the design of experiments that generated them. This approach, called ‘Knowledge Engineering from Experimental Design’ (KEfED, 10) attempts to construct data structures for experiments based on dependency relationships between the values of parameters and their accompanying measurements. Within KEfED, these dependency relationships are derived from the structure of the experimental protocol used to generate them.

The interaction between observation and interpretation has been previously formulated in cyclic models of scientific reasoning (see 11, 12). This methodology has formed the basis for the development of completely automated robotic systems that are capable of formulating scientific experiments, performing them and then interpreting their results to propose new experiments (13). Ultimately, this degree of automation is the aspiration goal of programs like Big Mechanisms: to develop computationally scalable methods to understand complex phenomena.

### Natural language processing studies of discourse analysis

Related work on discourse analysis of biological text has focused on different levels of textual granularity, representing different views on the key discourse moves contained within a text. Different approaches are used to identify assertions from text, either manually, automatically, or semi-automatically. Very often, these require discourse parsing as a first step. Marcu (14) automatically identifies Rhetorical Structure Theory relations (15) between elementary discourse units (edu’s). More recent systems attempting the same task include (16) and (17). The work of Teufel (18) focuses on finding so-called argumentative zones, which are defined as a (group of) sentences that have the same rhetorical goal. Teufel (18) identify six such zones, such as those defining ‘own’ vs. ‘other’ work; stating the background of a piece of work, or its results. Mizuta and Collier (19) identify similar though finer-grained zones in biological texts. Biber and Jones (20) define a collection of biological Discourse Units, and the respective Discourse Unit Type by various linguistic markers.

Using the XIP dependency parser (21, 22) aimed at detecting rhetorical metadiscourse functions that are attached to propositions in biology articles. The status of a proposition may be, e.g. that of a substantially new finding; the author may want to state that a particular solution is not known; a statement may serve as background knowledge; it may be a contradiction, hypothesis, or a new research tendency. A further body of work on event extraction identifies a ‘bio-event’, namely a representation of important facts and findings (e.g. 23) that exists at the level of a sentence or a few sentences. The CoreSC Ontology developed by Liakata et al. (24) defines a set of sentence-level classes that describe scientific investigations. In earlier work (e.g. 25, 26) we proposed a third level of granularity, that of discourse segments, which roughly corresponds to a clause. Using manual annotation methods, a taxonomy was identified to define the main discourse segments in biological text that correlated with grammatical and semantic markers such as verb form, tense, and modality markers. All three methods (bio-events, core-sc and discourse segment type) were compared and found to be compatible, yet subtly different; see Liakata et al., (27) for a side-by-side comparison.

### Applying discourse analysis to a biomedical research narrative

In our previous work, we noted that different research domains typically follow different narrative structures based on the procedural structure of their experimental protocols (1, 10, 28–30). Typically, articles in molecular biology construct their argument based on a number (as high as 20–40) individual small-scale experiments involving small numbers of assays with generally minimally differing design (1). In contrast, neuroanatomical studies describe a smaller number of representative sample cases from a set of experiments conforming to a single, relatively simple design. This is because data analysis is much more time consuming to perform than standardized biochemical assays (10, 28). By way of further contrast, studies of vaccines with non-human primates involve complex protocols with a relatively small number of individual cases due to the high value of individual experimental subjects (30).

This motivates the underlying purpose of our study, and the basis of our information extraction strategy for the Big Mechanisms program: to distinguish between statements concerned with (i) experimental methodology and findings, (ii) interpretative assertions based on those findings and (iii) contextual statements that motivate the need for the experiment in the broader narrative of the article as a whole (31).

We do this by combining our KEfED model with the clause-level taxonomy defined by De Waard and Pander Maat (32) that classifies each clause as belonging to one of seven: (i) Facts, (ii) Hypotheses, (iii) Problems, (iv) Goals, (v) Methods, (vi) Results and (vii) Implications, which mostly occur in a prespecified order. If we look at the typical narrative structure of a molecular biology article, authors typically write passages to construct a scientific argument, based on their experiments and use epistemic segments to provide the necessary discourse structure. This discourse structure is highly variable in general, but within the results sections of articles, the following archetypal example is illustrative of how an author may present their findings. Authors may first introduce a new semantic context for their experiments by describing ‘problem’ elements found in other work, or stating facts, results or implications from previous articles (identifiable by citations). They may describe the underlying goal (or hypothesis) of their experimental work, and to this point in the text, have mostly discussed interpretive concepts at the level of the conceptual model. Next, they turn to a description of their experimental experiences: the methods used to perform the experiment, which will usually include values of specific parameters that were set in the study design to contextualize the data findings. This is typically following by results, in which a combination of values of parameters, measurements, and immediate interpretive assertions may be given. After this—after showing the experimental motivation for their conceptual statements— the interpretations and conclusions generally appear: these are the global inferences that exist at the level of the overall conceptual framework/model, which we call here the experiment’s implications). De Waard (33) provides a more detailed description of the ‘realm transitions’ between conceptual model and physical experiment, and Tallis et al. (30) provide a discussion of the cyclic reasoning process between observations and interpretations in relation to the KEfED approach.

**Figure 1.**
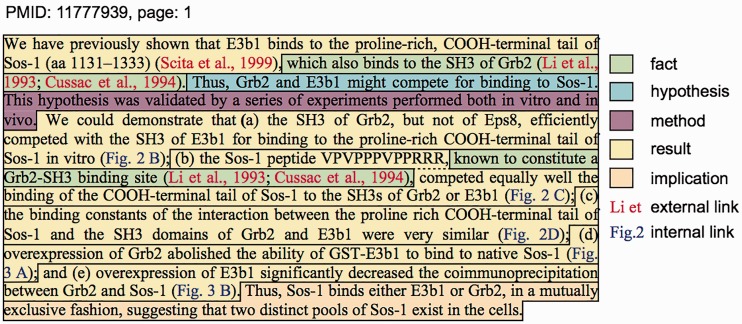
A typical passage from a primary research article describing experimental results (34) with added annotations describing discourse segment types, internal links to figures and external links to cited references.

Figure 1 shows a typical text passage from a scientific research article (34) from the PL corpus describing results from coprecipitation experiments designed to show how specific molecules compete in binding reactions. The figure shows a single paragraph where the authors provide some context by citing their previous work, stating some facts, and then providing a hypothesis. The authors then provide a very brief placeholder for the methods used (supported by the text of figure legends, not shown). They then describe results of five separate experiments in quick succession where each experiment is labeled by the each listed subfigure (2B, 2C, 2E, 3A, 3B). It is important to note that in this case, each of these subfigures denotes a separate experiment with quite different designs and data (involving the use of concentration gradients, time series or sophisticated use of the immunoprecipitation methodology). Finally, the author provides a unifying explanation of these results with an implication statement. Note that it is important to distinguish between the description of results in this article (denoted by the presence of an internal link to a figure) and the description of results from other studies (denoted by the presence of an external link expressed as citations).

### Previous work: BioCreative, protein–protein interactions, MINT, and INTACT

Automatically extracting aspects of experimental descriptions the basis of the community-wide BioCreative 2 (BC2) and 3 (BC3) competitions. These included evaluations on various aspects of protein–protein interactions (35, 36), including article detection, interaction pair extraction, interaction method extraction (IMS) and (D) retrieval of ‘interaction sentences’. Two BC2 teams and eight BC3 teams addressed the IMS subtask, where the aim was to extract the correct ontology code describing the interaction detection method (from the standard PSI-MI2.5 vocabulary, see (37)). The results were compared with manually curated codes for each article under various conditions (exact- and parent-match or micro-/macro-averages) across the 2 years. High-performing systems on this task included the Ontogene system’s approach in BC2 (38; F1 score = 0.65) and efforts in BC3 (36; F1 score = 0.55). It is important to note that the curation workflow of the BioCreative challenges centered first on the analysis of interpretive text describing interactions and only secondarily dealt with text on experimental evidence (see Figure 1 from (35)). Since the MINT database has been subsumed by the INTACT project (39), we repeated the key analysis from BC2 and BC3 with a focus detecting the experimental type derived from text found in the results section with both MINT and INTACT data.

## Materials and methods

The goals of the experimental work in this article are as follows. First, we intend to automatically detect the different types of discourse segments within passages from articles’ Results sections so that we might prioritize the most relevant text pertaining to different aspects of the narrative. Second, we seek to identify the type of experiment that is being referred to in the sentences describing results. Both of these intermediate results support the development of automated machine reading systems that thoroughly analyze all aspects of experimental research articles in this domain.

### The PL datum repository

The PL group is based at SRI International in Menlo Park CA and is a performer in the Big Mechanisms program. The system comprises an approach to modeling cellular signaling pathways based on ‘rewriting logic’ (6). The PL database is populated by the human curation of observable findings from each experimental article (where each entry is called a ‘datum’). Expert biocurators have also defined a broad classification of the different types of molecular biological assays used in the experiments annotated in the data set (documented online at http://pl.csl.sri.com/CurationNotebook/). Since this database is centered on the same distinction between interpretations and observations forming the core of our methodology, we based our initial information extraction work on this data. In total, the PL database contains ∼2000 articles of which 76 have been designated part of the open-access subset in the Pubmed Central (PMC) online library. These 76 articles provide the natural language data for the experimental work described here.

### Open access articles from the MINT and INTACT databases

The ‘Molecular Interaction’ (MINT) database is based on experimentally verified protein–protein interactions mined from the scientific literature by expert curators (40, 41). The full MINT dataset can be freely downloaded from http://mint.bio.uniroma2.it/mint/download.do. Within this collection, we were interested in the curator-driven annotation of experimental type denoted by Protein Standards Initiative Molecular Interaction XML Format (‘PSI-MI’) format that provides codes pertaining to the experimental types used to detect MINTs (see http://www.psidev.info/index.php?q=node/60). At present, MINT contains data from 5554 articles. We downloaded information describing human protein–protein interaction experiments from 2808 of them. Of these articles, we identified 175 articles that were in the open-access PMC subset, which we then used in the work described here. We downloaded the full database of annotations from the INTACT repository (which includes all of MINT with changes in the translation of the data, http://www.ebi.ac.uk/intact/downloads). This consisted of 32 678 interaction reports from 14 009 published articles, of which 1063 are available as open access. In both cases, the starting point that enabled our classification analysis was the specific sub-figure associated with each experiment being annotated.

### Data acquisition and preprocessing

The raw textual data for processing was extracted from the relevant articles in the Open Access Subset of PMC with the ‘nxml2txt’ tool (https://github.com/spyysalo/nxml2txt) as part of the UimaBioC core library (42). UimaBioC converts the bibliographic annotations present in the XML-formatted articles from PMC (formatting, section-based headers, hyperlinks etc.) into readable annotations in the BioC format for subsequent processing and reuse. As a parallel thread, we applied the REACH system from the Computational Language Understanding Lab at the University of Arizona, which employs a pattern-based entity and event tagger for these articles (43). We extracted the text of the results sections of the abovementioned 76 articles as training data for the subsequent task of classifying discourse segments.

### Text annotation

Our definition of discourse segment type broadly follows that in (32), and we maintain an online annotation manual to track notes. Initially, we used the BioScholar digital library system (44) to manually annotate passages of text pertaining to individual experiments based on their discourse segment types. This in-house software stores annotations based on overlays constructed over the rendered pages of PDF files (thus making the annotation task relatively intuitive for biological curators). We manually curated the text of results sections based on the precise sub-figure the text was referring to (e.g. ‘1A’, ‘1B’ etc.) and then assigned an epistemic type label (such as hypothesis, fact etc.) to each chunk. This provided a core set of texts to provide an initial data set, which we then augmented. Given the set of 76 open access articles provided by PL, we extracted the results sections from these articles, split the sentences into clauses using parsing tools from the NLTk Python toolkit (45), and had annotators manually assign each clause one of the seven discourse segment label types or the label ‘none’ (in cases when a clause was a subject header).

### Machine learning discourse segment types

We developed a machine learning system for automatically assigning discourse segment types to the statements derived from research articles. As a baseline, we configured a Support Vector Machine (SVM) in a seven-way classifier (i.e. a combination of seven one-vs.-all classifiers) designed to label each clause based on our seven discourse segment types (namely, ‘fact’, ‘hypothesis’, ‘problem’, ‘goal’, ‘method’, ‘result’ and ‘implication’). To train the classifier, we used the previously described manually annotated training examples. The clauses were then represented as feature vectors based on the following scheme: (i) ‘Part-of-speech tags’: We applied the Stanford POS tagger (46) to the clauses and added the part of speech tags to the feature vector. (ii) ‘Verbs and adverbs’: Based on the POS tags, we added all the verbs and adverbs from the clause to the feature vector. These words are generally indicative of the statement type. (iii) ‘Figure reference or a citation’: If the clause contained a pointer to a figure in the article, or a citation, we added this information its feature vector since this usually indicates that the statement should be classified is a ‘result’. (iv) ‘Lexicon’: We created a small list of words and phrases indicative of the statement classification and added the presence (or absence) of these phrases in the clause to the feature vector. For example, if a statement contained ‘possible’, the statement would typically be classified as a ‘hypothesis’, while if the statement contained ‘data not shown’, the statement would likely be a ‘result’.

Additionally, we observed that discourse segments in results sections often followed a sequence approximating (i) fact/hypothesis/problem, (ii) goal, (iii) method, (iv) result and (v) implication. We therefore trained a Conditional Random Field (CRF) (47) and compared its performance to the SVM classifier, expecting an improvement since the CRF is also sensitive to patterns in label ordering. For this, we used part of speech tags, the identities of verbs and adverbs, presence of figure references and citations, and hand-crafted lexicon features. For example, words like ‘demonstrate’ and ‘suggest’ indicate implication, and phrases like ‘data not shown’ indicate results that indicate specific discourse types. The choice of features was based on standard feature engineering practices to identify the most salient. Small-scale experiments with larger lexicons did not improve performance and validated this selection of restricted feature set. Beyond this, we did not perform an exhaustive comparative study of the contribution of different feature sets to performance.

### Automatic partitioning of experiment descriptions

To create a database of experiment descriptions, we first need to assemble all the text’s statements about each experiment. Our core assumption is that each figure corresponds to one experiment, and within it each sub-figure to its sub-experiment variations. In a small manual evaluation of how figures relate to different experiments, we found that only 2 experiments from 128 separate experiments in over 10 articles involved more than one Figure. In total, 76% of all sub-figures referred to distinct experiments (based on whether a different experimental subject was processed under a different protocol), and the types of experiments within a single figure were usually closely related.

Fortunately, the linkage between figures and experiments within the training data is contained in the manually curated data sets from INTACT, MINT and PL (who generate their data according to their own internal standards, see http://pl.csl.sri.com/CurationNotebook/index.html and http://www.imexconsortium.org/curation). We simply adopted this data as a Gold Standard consistent with standard practice when professionally curated data are available.

### Automatic classification of experiment types

Each of the PL Datum entries has been previously annotated by expert curators with (i) the subfigure from which it originated and (ii) the type of experiment performed under the PL annotation scheme (see http://pl.csl.sri.com/CurationNotebook/pages/Assays.html). There are 33 separate ‘assay types’ used to denote different high-level classes of frame representation, including coprecipitation (denoted by the code: ‘copptby’), phosphoryation (‘phos’), colocalization (‘colocwith’), acetylation, sumoylation, ubiquitination (‘ubiq’), cellular location (‘locatedin’) and others. These annotations denote a complex classification of the type of knowledge being generated by each of the annotated experiments and correspond in more depth to the combination of multiple steps described in the text describing the methods used in the context of each sub-figure.

In contrast, the articles downloaded from the open-access dataset of either the MINT or INTACT database use PSI-MI 2.5 codes, which somewhat overlap the experimental type codes used in the PL database, especially those pertaining to ‘coimmunoprecipitation’ as a core methodology. These articles offer a more in-depth view of experimental designs pertaining to studies of protein–protein interactions and binding but ignore other functional aspects of molecular biology that are captured in PL. In both cases, we applied machine learning systems that could automatically predict the type of experiment from the relevant text extracted from the results section and figure legend.

We used a simple heuristic to match text passages from the body of research articles to sub-figures (simply based on matching paragraphs as a whole to the sub-figures referenced in them). This was problematic since there can be multiple figure references within a passage and each figure reference could be classified as a different experiment type. Within the PL dataset, of the available 372 passages, we identified 114 passages that contained more than one basic code, which we removed from the classification experiments. We used a training set based on 258 passages and evaluated performance based on mean accuracy and weighted F1 measures from 5-fold cross validation with 24 different target classes. We repeated this process for articles from the MINT database and of the 359 passages we found from experimental text referring to figures, 136 of these passages were discarded due to referring to multiple types of experiments. For MINT, we used 223 passages (=359−136) with 9 classes (‘pull down’, ‘anti tag coimmunoprecipitation’, ‘anti bait coimmunoprecipitation’, ‘x-ray crystallography’, ‘fluorescence microscopy’, ‘coimmunoprecipitation’, ‘two hybrid’, ‘surface plasmon resonance’, ‘imaging technique’). The INTACT database provided a more extensive set of PSI-MI 2.5 interaction methods annotations linked to subfigures. We restricted the classification task to the top 5, 10 and 15 most frequent experimental codes (mirroring the approach used in (38)). We ran classification experiments on all available data following the same simplified method of selecting paragraphs with well-defined figure references to establish baseline performance for this classification task.

Additionally, to examine the utility of using only text from the figure caption, we developed simple patterns to identify well-defined sub-figure caption sentences that begin with a single letter in parentheses (‘(A)…’ etc.) as text to be processed by our classifier and compared this to our methods based on text in the narrative. We applied as baseline a simple term-based classifier, using terms identified for each class by tf.idf (term frequency/inverse document frequency) scores, in a 5-fold cross validation.

## Results

### Linking experiments to figures

A key element of this work is the linkage between individual subfigures and their underlying experiments. This linkage arises from core design decisions in both the PL and MINT data sets, and we performed a small-scale manual evaluation as a part of this study. To consider the question of how text describing figures in results sections should be evaluated as originating from separate experiments, we examined 10 articles from the PL corpus and judged whether experiments from two figures referred to the same experiment based on whether the data shown were derived from the same underlying experimental subjects undergoing to the same protocol. These 10 articles contained 50 figures and 213 subfigures. We evaluated that there were 128 separate experiments performed and that the number of subfigures per experiment ranged from 1 to 23. In only two cases from this sample set did a single experiment involve more than one figure (both of these cases arose from the same article). 76% (98/128) of experiments delineated in this way were reported in only one subfigure and 88% (113/128) were reported in either one or two subfigures. This is a small sample intended only to act as justification for the methodology we have adopted and would benefit from a larger scale biocuration effort.

### Machine classification of discourse segment type

Our annotated dataset for statement types included 20 articles, containing 3600 clauses. We used 200 of the clauses as a held-out test set to measure the performance of our classifier on unseen data. We trained our statement classifier on the remaining data. Table 1 summarizes ground truth, predictions, precision, recall and F1 scores on the test set for our CRF system (also see Supplementary Materials).

**Table 1.** Number of ground truth assertions and predictions associated with precision, recall and F1 scores for discourse segment type classification based on CRF analysis.

Statement type	Ground truth	Prediction	Correct predictions	Precision	Recall	F1
Problem	2	2	0	0.00	0.00	0.00
Fact	53	30	14	0.47	0.26	0.34
Hypothesis	36	17	12	0.71	0.33	0.45
Goal	30	26	18	0.69	0.60	0.64
Method	98	90	66	0.73	0.67	0.70
Result	182	216	147	0.68	0.81	0.74
Implication	44	64	31	0.48	0.70	0.57
**Weighted F1**						**0.63**

We found that discourse segment type classification performed well for ‘results’ statements, at an F1 score of 0.78. Other discourse segment types were detected with varying levels of accuracy depending on the annotation frequency for each one in the corpus. At present, our sample size is not large enough to detect the rarer discourse segment tags such as ‘problem’, ‘fact’ or ‘hypothesis’ (which is not surprising since these statements are much rarer than descriptions of methods or results in ‘Results’ sections).

In this study, our focus was centered on unpacking the discourse structure used by authors in Results sections to narrate over their experimental findings as a sequence of well-defined discourse types. We expect that the discourse structure of other sections will include more problem, fact, and hypothesis statements, and used in various orders, thus less likely to conform to the structured order we were studying in results sections. We present a confusion matrix for this analysis in Table 2.

**Table 2.** Confusion matrix for discourse segment type classification

	**Predicted values**								
	Counts	Problem	Fact	Hypothesis	Goal	Method	Result	Implication	
**Ground truth values**	Problem	0	0	0	0	0	2	0	2
	Fact	1	14	2	2	2	26	6	53
	Hypothesis	0	2	12	5	3	6	8	36
	Goal	0	0	0	18	9	2	1	30
	Method	0	4	0	1	66	26	1	98
	Result	1	7	2	0	8	147	17	182
	Implication	0	3	1	0	2	7	31	44
			2	30	17	26	90	216	64	

The primary confounding classification mistake in classifying statements as ‘fact’ discourse segment types is that they are mistaken for ‘result’ statements. Given that the distinguishing feature between facts and results is based on the information source that they are each derived from (facts are presented without attribution to a source, but results are derived from measurements of the referenced experiments), this is hardly surprising. Similarly, confusion between results and implications had an impact of classification performance between those types as well.

### Machine classification of experiment type

Table 3 shows data pertaining to ground truth, predictions, precision, recall and F1 scores for classification of experimental passages referenced in two databases with two different classification schemes (see supplemental data for performance across different classifiers). The analysis shows generally good performance with an overall weighted F1 score of 0.63 for PL. The best performance with the INTACT dataset was given by the text from well-defined figure captions (see ‘Materials and Methods’ section above) was a F1 score of 0.71 for 581 experiments. Given that these results are the product of simple generic features without any tailored specialization for our specific tasks, we expect that future work will be able to improve considerably upon this baseline. We were concerned that these data were dominated by the presence of a single very frequent type (such as MI:0019, ‘coimmunoprecipitation’), which would skew performance. We therefore computed a baseline F1 score for the situation where all experiments were classified as being of the same (i.e. most frequent) type. In all cases, performance of our tools exceeded this baseline.

**Table 3.** Summary table of number of experimental cases, baseline F1 scores and experimental F1 scores for experiment type classification from PL, MINT and INTACT datasets

	**No. experiments**	**Baseline F1-score**	**F1-score**
PL, all data, narrative	258	0.07	0.63
INTACT, top 5 leaf categories, narrative	1160	0.13	0.44
INTACT, top 5 merged categories, narrative	1290	0.29	0.54
INTACT, top 10 merged categories, narrative	1507	0.26	0.50
INTACT, top 5 merged categories, captions	**581**	**0.42**	**0.71**
INTACT, top 10 merged categories, captions	662	0.34	0.67
MINT, top 7 categories, narrative	221	0.24	0.58

The confusion matrix for the best performing system (INTACT data, top five merged categories, classification based on figure caption text) is shown in Table 4. This shows the strong influence of the most likely experimental category as a source of inaccurate predictions. This is consistent with how common coimmunoprecipitation experiments are in the literature to detect MINTs.

**Table 4.** Confusion matrix for experiment type classification

Gold/prediction	MI:0018	MI:0019	MI:0096	MI:0416	MI:0663	
**MI:0018**	25	29	3	0	0	57
**MI:0019**	0	321	9	0	2	332
**MI:0096**	1	53	71	0	0	125
**MI:0416**	0	26	0	4	4	34
**MI:0663**	0	20	0	3	10	33
	26	449	83	7	16	

## Discussion/conclusions

A core concept underlying our work is that primary research publications contain conclusions typically based on a discrete number of experiments that are described in detail as ‘results’. Within many curated bioinformatics systems (such as the Gene Ontology, 48), these experiments and their data play only a supporting role as ‘evidence’ for interpretive claims that form the primary focus of the resource (see 49 for evidence codes of GO, or (49) for the evidence representation of the BioPax representation).

We have begun work to automate the process of extracting experimental observations from articles based on subdividing the text of articles into relatively compact discourse elements pertaining to a single experiment. Our preliminary results show some success in using simple supervised machine learning to classify these discourse elements by experimental type, allowing one to distinguish different types of assertion from the experimental narrative. This lays the groundwork for targeted, structured information extraction systems based on multi-sentence extraction in the future.

We observed, while annotating documents with discourse types that authors use a recurring sequence, consisting of only a few sentences within a paragraph, to present new data and construct an argument. Typically, they provide background knowledge, state hypotheses, introduce goals and methods, describe results and then postulate implications within the text of the results section. In our experiments, we were therefore not surprised to find that sequence classifiers using CRFs performed better than other methods such as SVMs or a simple tf.idf-weighted term-matching baseline. This suggests that there are implicit cycles of scientific reasoning in the text that permit authors to incorporate experimental evidence for claims into the narrative argument of a study. CRF techniques have been a mainstay technique for sequence labeling work for 15 years but are now being superseded by deep learning methods that require less painstaking feature engineering analysis (51). We therefore expect to be able to outperform this early work with neural network methods that are able to discover optimal feature sets automatically.

Studies of argumentation structure in biomedical articles used a very similar annotation scheme to our discourse labeling method as part of the CRAFT full-text annotation effort (52). This could potentially provide extensive additional training data for subsequent work (although these annotations were based on Mouse genetics studies, a different target domain from the cancer pathway articles of interest to our work).

We previously argued in a position article that rather than focusing efforts only on the complexities of extracting semantically structured interpretive events from text, it may be more tractable and reliable to focus on extracting the data and observations directly gathered from experiments (1). We here describe preliminary results that instantiate and test this strategy.

Within this article, we simplified the issue of delineating the passages pertaining to specific experiments by considering entire paragraphs as the source of data used by classifiers. Naturally, a finer-grained delineation of the narrative pertaining to individual experiments warrants deeper investigation and could be a valuable area of study in its own right. We previously examined this question in a different field of biomedicine (neuroanatomy, 53). From this, we expect each separate experimental (sub)domain to use different narrative approaches to explain and unpack their results. Thus, heuristic methods used to frame a machine-reading approach in the field of molecular biology should not be applied generally across other domains.

The most common type of experimental evidence available in our training corpus pertains to coimmunoprecipitation studies: a staple of studies into MINTs and how molecules bind to each other. The MINT databases complete list of codes derived from the PSI-MI 2.5 controlled vocabulary defined 93 possible types of binding assay (50), of which we use a subset in our studies here. This provides a rich working space for modeling and investigating the substructure of different experiments of this type, since the way that assertions would be constructed from data would depend on way any given protocol is parameterized and structured. The INTACT dataset increased the quantity of data available for this classification task. Of particular interest is the reliable performance improvement we observed based on using only the text from the figure caption to denote the type of experiment. This text is easier to extract and identify than delineating the exact span of relevant text in the narrative of a article. Our results suggest that the figure caption might act as a more accurate and more practical predictor for experimental type than narrative text in the results sections of articles. How we represent experimental evidence in bioinformatics infrastructure is an open research question (54). Being able to detect experimental type reliably could accelerate curation and assist more sophisticated representations of evidence to support claims in databases.

Ontology support for different modeling approaches to experimental design has been addressed by multiple efforts including the Open Biomedical Ontology’s ‘Ontology of Biomedical Investigation’ (55) and the Biological Assay Ontology (56) as well as multiple ‘Minimum Information’ checklist representations (57). A confounding issue of leveraging these representations into a text mining biocuration toolset is the underlying inherent multilevel complexity of describing scientific protocols, which may be alleviated by developing templates under a methodology such as KEfED (30) that could also refer to grounded, standardized ontological terms where necessary.

In this article, we present the initial stages of developing a strategic idea, designed to promote a machine reading method for biocuration that examines the experimental context in detail. Even interpretive claims about mechanisms must themselves be derived from a chain of reasoning driven by experimental evidence at every stage, and our work is concerned with deepening the structured representation of evidence that may be extracted from the text of primary research articles. This work is intended to support the efforts of other machine reading groups by indicating passages that contain extracted data that have assumed to be ‘facts’ but were actually written as ‘hypotheses’ by the original authors. Our work represents and extracts such evidence at a much finer granularity than is currently supported computationally. We continue to seek general principles that could be applied across different domains based on general practices of describing experimental results and reduced to practice easily and straightforwardly through the use of standard machine learning tools.

## Supplementary Material

Supplementary Data

## References

[1] Burns G.A.P.C. Chalupsky H. (2014). “Its All Made Up” - Why we should stop building representations based on interpretive models and focus on experimental evidence instead. In Discovery Informatics: Scientific Discoveries Enabled by AI, (Quebec City, Quebec)

[2] Hucka M. Finney A. Sauro H.M., (2003) The systems biology markup language (SBML): a medium for representation and exchange of biochemical network models. Bioinformatics, 19, 524–531. 12611808 10.1093/bioinformatics/btg015

[3] Demir E. Cary M.P. Paley S., (2010) The BioPAX community standard for pathway data sharing. Nat. Biotechnol., 28, 935–942. 20829833 10.1038/nbt.1666 PMC3001121

[4] Hayes W. (2015) OpenBel Framework Release 3.0.0 (build 2015-07-28), https://github.com/OpenBEL/openbel-framework/releases/tag/3.0.0_build20150728

[5] Korkut A. Wang W. Demir E., (2015) Perturbation biology nominates upstream-downstream drug combinations in RAF inhibitor resistant melanoma cells. eLife, 4. 10.7554/eLife.04640 PMC4539601 26284497

[6] Eker S. Knapp M. Laderoute K., (2002). Pathway Logic: Executable Models of Biological Networks In Fourth International Workshop on Rewriting Logic and Its Applications (WRLA’2002), Elsevier, Amsterdam.

[7] Danos V. Feret J. Fontana W., (2008). Rule-based modelling, symmetries, refinements In Fisher J. (ed). Formal Methods in Systems Biology, Springer, Berlin Heidelberg, pp. 103–122.

[8] Lopez, C.F., Muhlich, J.L., Bachman, J.A., and Sorger, P.K. (2013). Programming biological models in Python using PySB. *Mol Sys Biol*, *9*, 646. 10.1038/msb.2013.1 PMC3588907 23423320

[9] Slater T. (2014) Recent advances in modeling languages for pathway maps and computable biological networks. Drug Discov. Today, 19, 193–198. 24444544 10.1016/j.drudis.2013.12.011

[10] Russ T. Ramakrishnan C. Hovy E., (2011) Knowledge engineering tools for reasoning with scientific observations and interpretations: a neural connectivity use case. BMC Bioinformatics, 12, 351. 21859449 10.1186/1471-2105-12-351 PMC3176268

[11] Soldatova L.N. Rzhetsky A. (2011) Representation of research hypotheses. J. Biomed. Semant., 2, S9. 10.1186/2041-1480-2-S2-S9 PMC3102898 21624164

[12] Clark T. Kinoshita J. (2007) Alzforum and SWAN: the present and future of scientific web communities. Brief Bioinform., 8, 163–171. 17510163 10.1093/bib/bbm012

[13] King R.D. Rowland J. Aubrey W., (2009) The Robot Scientist Adam. Computer, 42, 46–54.

[14] Marcu, D. (1999). A decision-based approach to rhetorical parsing. In Proceedings of the 37th Annual Meeting of the Association for Computational Linguistics on Computational Linguistics, (Association for Computational Linguistics), pp. 365–372.

[15] Mann, W.C., and Thompson, S.A. (1987). Rhetorical structure theory: A theory of text organization (University of Southern California, Information Sciences Institute).

[16] Ji Y. Eisenstein J. (2014). Representation learning for text-level discourse parsing. In *Proceedings of the 52nd Annual Meeting of the Association for Computational Linguistics (ACL)*, Baltimore, Maryland.

[17] Joty S. Carenini G. Ng R. (2015) Codra: A novel discriminative framework for rhetorical analysis. Comput. Linguistics, 41, 385–435.

[18] Teufel S. (1999) Argumentative zoning: information extraction from scientific text. *Ph.D. Thesis*, University of Edinburgh, 1999

[19] Mizuta, Y., and Collier, N. (2004). Zone Identification in Biology Articles As a Basis for Information Extraction. In Proceedings of the International Joint Workshop on Natural Language Processing in Biomedicine and Its Applications, (Stroudsburg, PA, USA: Association for Computational Linguistics), pp. 29–35.

[20] Biber D. Jones J.K. (2005) Merging corpus linguistic and discourse analytic research goals: discourse units in biology research articles. Corpus Linguistics Linguistic Theory, 2, 151–182.

[21] Aït-Mokhtar S. Chanod J.-P. Roux C. (2002) Robustness beyond shallowness: incremental dependency parsing. Nat. Lang. Eng., 8, 121–144.

[22] Sándor Á. (2007) Modeling metadiscourse conveying the author's rhetorical strategy in biomedical research abstracts. Revue Française de Linguistique Appliquée, 200, 97–109.

[23] Thompson P. Nawaz R. McNaught J. Ananiadou S. (2011) Enriching a biomedical event corpus with meta-knowledge annotation. BMC Bioinformatics, 12, 393. 21985429 10.1186/1471-2105-12-393 PMC3222636

[24] Liakata, M., Teufel, S., Siddharthan, A., Batchelor, C.R. (2010). Corpora for the Conceptualisation and Zoning of Scientific Papers. *Language Resources and Evaluation Conference (LREC)*. Malta

[25] Liakata, M., Saha, S., Dobnik, S., Batchelor, C., and Rebholz-Schuhmann, D. (2012). Automatic recognition of conceptualization zones in scientific articles and two life science applications. Bioinformatics, 28, 991–1000. 10.1093/bioinformatics/bts071 PMC3315721 22321698

[26] De Waard A. (2007), A pragmatic structure for research articles. In *ICPW ’07 Proceedings of the 2nd international conference on Pragmatic web*, pp, 83–89.

[27] De Waard A. Pander Maat H. (2012). Epistemic modality and knowledge attribution in scientific discourse: a taxonomy of types and overview of features. *Proceedings of the Workshop on Detecting Structure in Scholarly Discourse*, pp. 47–55.

[28] Burns G.A.P.C. Turner J.A. (2013). Modeling functional Magnetic Resonance Imaging (fMRI) experimental variables in the Ontology of Experimental Variables and Values (OoEVV). Neuroimage, 82, 662–70. 10.1016/j.neuroimage.2013.05.024 PMC4474486 23684873

[29] Tallis M. Thompson R. Russ T.A. Burns G.A.P.C. (2011) Knowledge synthesis with maps of neural connectivity. Front Neuroinform., 5, 24. 22053155 10.3389/fninf.2011.00024 PMC3205380

[30] Tallis M. Dave D. Burns G.A. (2012). Preliminary meta-analyses of experimental design with examples from HIV vaccine protection studies. In *Discovery Informatics Symposium DIS2012*, Arlington, VA.

[31] Gama-Castro S. Rinaldi F. López-Fuentes A., (2014) Assisted curation of regulatory interactions and growth conditions of OxyR in E. coli K-12. Database, 2014, 1–13. http://doi.org/10.1093/database/bau049 10.1093/database/bau049 PMC4207228 24903516

[32] De Waard A. Pander Maat H.L.W. (2012b) Verb form indicates discourse segment type in biological research papers: experimental evidence. J. Engl. Acad. Purp., 11, 357–366. http://dx.doi.org/10.1016/j.jeap.2012.06.002

[33] De Waard A. (2010), Realm traversal in biological discourse: from model to experiment and back again, *Workshop on Multidisciplinary Perspectives on Signalling Text Organisation (MAD 2010)*, March 17–20, 2010, Moissac, France.

[34] Innocenti M. Tenca P. Frittoli E., (2002) Mechanisms through which Sos-1 coordinates the activation of Ras and Rac. J. Cell Biol., 156, 125–136. 11777939 10.1083/jcb.200108035 PMC2173577

[35] Krallinger M. Leitner F. Rodriguez-Penagos C. Valencia A. (2008) Overview of the protein-protein interaction annotation extraction task of BioCreative II. Genome Biol., 9, S4. 18834495 10.1186/gb-2008-9-s2-s4 PMC2559988

[36] Krallinger M. Vazquez M. Leitner F., (2011) The Protein-Protein Interaction tasks of BioCreative III: classification/ranking of articles and linking bio-ontology concepts to full text. BMC Bioinformatics, 12, S3. 22151929 10.1186/1471-2105-12-S8-S3 PMC3269938

[37] Orchard S. Montecchi-Palazzi L. Hermjakob H. Apweiler R. (2005) The use of common ontologies and controlled vocabularies to enable data exchange and deposition for complex proteomic experiments. Pac. Symp. Biocomput. 2005, 186–196. 15759625

[38] Rinaldi F. Kappeler T. Kaljurand K., (2008) OntoGene in BioCreative II. Genome Biol., 9, S13. 18834491 10.1186/gb-2008-9-s2-s13 PMC2559984

[39] Orchard S. Ammari M. Aranda B., (2014) The MIntAct project–IntAct as a common curation platform for 11 molecular interaction databases. Nucleic Acids Res., 42, D358–D363. 24234451 10.1093/nar/gkt1115 PMC3965093

[40] Zanzoni A. Montecchi-Palazzi L. Quondam M., (2002) MINT: a Molecular INTeraction database. FEBS Lett., 513, 135–140. 11911893 10.1016/s0014-5793(01)03293-8

[41] Chatr-Aryamontri A. Ceol A. Palazzi L.M., (2007) MINT: the Molecular INTeraction database. Nucleic Acids Res., 35, D572–D574. 17135203 10.1093/nar/gkl950 PMC1751541

[42] Burns G.A. (2015a) The UimaBioC Software Library, release v0.1 https://doi.org/10.5281/zenodo.32637

[43] Surdenau M. (2015) The REACH Application, v1.1.0-SNAPSHOT, https://github.com/clulab/reach/releases/tag/1.1.0-SNAPSHOT

[44] Burns G.A. (2015b) The BioScholar Web Application, v1.1.5-SNAPSHOT, https://doi.org/10.5281/zenodo.32750

[45] Bird S. (2015) The NLTk Software Library, release 3.1, https://github.com/nltk/nltk/releases/tag/3.1

[46] Toutanova K. (2003) Feature-rich part-of-speech tagging with a cyclic dependency network. *Proceedings of the 2003 Conference of the North American Chapter of the Association for Computational Linguistics on Human Language Technology-Volume 1*. Association for Computational Linguistics, Sapporo, Japan.

[47] Lafferty J. McCallum A. Pereira F. (2001). Conditional random fields: probabilistic models for segmenting and labeling sequence data. In *Proceedings of the International Conference on Machine Learning*, Williamstown, MA, USA.

[48] Ashburner M. Ball C.A. Blake J.A., (2000) Gene ontology: tool for the unification of biology. The Gene Ontology Consortium. Nat. Genet., 25, 25–29. 10802651 10.1038/75556 PMC3037419

[49] GO Consortium. (2015) Guide to GO Evidence Codes. Available at http://www.geneontology.org/GO.evidence.shtml (date last accessed October 2015).

[50] Kerrien S. Orchard S. Montecchi-Palazzi L., (2007) Broadening the horizon–level 2.5 of the HUPO-PSI format for molecular interactions. BMC Biol., 5, 44. 17925023 10.1186/1741-7007-5-44 PMC2189715

[51] Graves A. (2012). Supervised Sequence Labelling with Recurrent Neural Networks, Springer-Verlag, Berlin Heidelberg.

[52] White E. Cohen K.B. Hunter L. (2011). The CISP annotation schema uncovers hypotheses and explanations in full-text scientific journal articles. In *Proceedings of BioNLP 2011 Workshop*, Portland, Oregon, USA: Association for Computational Linguistics, pp. 134–135.

[53] Feng D. Burns G. Hovy E. (2007). Extracting data records from unstructured biomedical full text. In *The Joint Meeting of Conference on Empirical Methods in Natural Language Processing and Conference on Computational Natural Language Learning (EMNLP-CoNLL 2007)*, Prague, Czech Republic.

[54] Chibucos M.C. Mungall C.J. Balakrishnan R., (2014) Standardized description of scientific evidence using the Evidence Ontology (ECO). Database, 2014, bau075 http://doi.org/10.1093/database/bau075 25052702 10.1093/database/bau075 PMC4105709

[55] Brinkman R.R. Courtot M. Derom D., (2010) Modeling biomedical experimental processes with OBI. J. Biomed. Semant., 1, S7. 10.1186/2041-1480-1-S1-S7 PMC2903726 20626927

[56] Visser U. Abeyruwan S. Vempati U., (2011) BioAssay Ontology (BAO): a semantic description of bioassays and high-throughput screening results. BMC Bioinformatics, 12, 257. 21702939 10.1186/1471-2105-12-257 PMC3149580

[57] Taylor C.F. Field D. Sansone S.A., (2008) Promoting coherent minimum reporting guidelines for biological and biomedical investigations: the MIBBI project. Nat. Biotechnol., 26, 889–896. 18688244 10.1038/nbt.1411 PMC2771753

