# Supplementary material for: Automated detection of discourse segment and experimental types from the text of cancer pathway results sections
Source: Database (Oxford). 2016 Aug 31;2016:baw122. doi: 10.1093/database/baw122 (PMC5006090; doi:10.1093/database/baw122)
Supplement: Supplementary Data [file supp_2016_baw122_index.html]

Automated detection of discourse segment and experimental types from the text of cancer pathway results sections — Supplementary Data 

# Automated detection of discourse segment and experimental types from the text of cancer pathway results sections

## Supplementary Data

files

- Supplementary Data - zip file
